# Supplementary material for: Chemical modulation of gut bacterial metabolism induces colanic acid and extends the lifespan of nematode and mammalian hosts
Source: PLoS Biol. 2025 Nov 11;23(11):e3002749. doi: 10.1371/journal.pbio.3002749 (PMC12604771; doi:10.1371/journal.pbio.3002749)
Supplement: Attachment — Submitted filename: Point-by-point Response.pdf [file pbio.3002749.s011.pdf]

Reviewer 1

This manuscript investigates a novel approach to modulating gut microbiota to enhance host longevity through chemical induction of bacterial metabolites. Specifically, the study explores how low doses of the antibiotic cephaloridine can induce the production of colanic acid (CA) in Escherichia coli (E. coli), which has been previously shown to extend the lifespan of the model organism Caenorhabditis elegans (C. elegans). The authors demonstrate that cephaloridine, at sub-minimum inhibitory concentrations, triggers the overproduction of CA by activating the cps operon in E. coli, even at temperatures above 30°C, which typically inhibit CA production. They extend their findings to the mammalian system by showing that low-dose cephaloridine can induce CA production in the mouse gut, suggesting potential translational relevance. The study also uncovers a novel mechanism for CA induction, involving the membrane-bound histidine kinase ZraS, independent of the RcsC-RcsD two-component system traditionally associated with CA biosynthesis.

While the study is well-conducted and presents intriguing findings, the manuscript's message could be misleading or incomplete in certain aspects.

Response: We appreciate that the reviewer finds our work being well-conducted and intriguing and provides suggestion to further improve the clarity of the manuscript.

Unclear Role of CA in Mice:

The manuscript does not adequately address the role of CA in mice, which is a significant gap considering that the study aims to explore the translational potential of CA induction from a microbial metabolite to a mammalian system. The authors need to clarify what is known about CA's effects in mammals, especially in the context of host longevity and health.

Response: We appreciate the reviewer's suggestions to investigate the role of CA in mice. In response, we orally supplemented low-dose cephaloridine alongside wild-type MG1655 *E. coli* to 1-year-old mice for six months and collected blood samples before and after the treatment. It is well established that LDL and insulin levels gradually increase with age in mice, due to aging-related metabolic dysfunctions. Consistently, we observed an age-related increase in LDL levels in male mice, whereas insulin levels increased in female mice between 12 and 18 months of age (new Figure 2H, 2I). Notably, these age-related increases were attenuated by the low-dose cephaloridine treatment (new Figure 2H, 2I). These new findings suggest cephaloridine-induced CA production mitigates age-related metabolic dysfunction in mice.

To further examine the possible role of CA induction in human longevity, we examined the presence of *cps* operon, which is responsible for CA production, in the Unified Human Gastrointestinal Genome (UHGG) database. We detected the existence of the *cps* operon in over 5000 genomes across 47 species, including *Escherichia, Salmonella, Enterobacter*, *Citrobacter* and others (new Figure 1A). Furthermore, we compared the presence of the *cps* operon between centenarians and their elderly controls using two publicly available human centenarian gut microbiome datasets (PRJNA675598, PRJEB25514). We found that the read count of the *cps* operon was significantly higher in centenarians compared to controls in the Japanese gut microbiome dataset (PRJNA675598), which includes more than 100 individuals in each group ($p=0.015$, new Fig. 1B). A similar trend was also observed using the Italian gut microbiome

dataset (PRJEB25514; *p=0.087*), although it did not reach statistical significance, likely due to the smaller sample size, with only ~20 individuals in each group (new Fig. 1C). These analyses revealed a potential correlation between the CA overproduction and human longevity.

Together, these findings support the beneficial effect of CA in mammalian systems and the translational potential of cephaloridine in promoting host health during aging. These new results have been included in the revised manuscript. We thank the reviewer for his/her valuable suggestions.

State-of-the-Art in Microbial Metabolic Pathway Activation:
The introduction lacks sufficient detail on the current understanding of drug-induced activation of microbial metabolic pathways in both commensals and pathogens. This background is crucial for contextualizing the novelty and significance of the study's approach.

Response: We thank the reviewer for the suggestion. We have included a new section (line 50-61) to introduce the current knowledge on drug-microbiota interaction with an emphasis on drug-induced activation of microbial metabolic pathways in both commensals and pathogens.

"The interaction between drugs and gut microbiota plays a crucial role in a variety of biological processes. One such process is the xenobiotic effect, where the microbiota converts prodrugs into active or inactive forms, thereby altering their bioavailability and bioactivity [PMID: 28642381]. Conversely, exposure to pharmaceuticals can influence metabolic pathways within the microbiota by modifying microbial community composition and regulating enzyme activities. Studies in both mice and humans have demonstrated that gastrointestinal exposure to various drugs leads to changes in the composition of the gut microbiota [PMID: 26701907] [PMID: 36894983] [PMID: 26719299]. Commonly used medications, such as proton pump inhibitors, metformin, antibiotics, and laxatives, have been linked to alterations in microbial metabolic pathways [PMID: 31953381]. However, the precise mechanisms underlying these drug-induced changes, as well as their functional consequences on gut physiology and host health, remain poorly understood."

The manuscript's intention to suggest a potential beneficial effect of CA in the host might be premature, given the limited evidence presented for its role in mammals. The most compelling part of the study is the discovery of a non-canonical mechanism for CA induction via cephaloridine. However, if the focus shifts solely to this mechanistic insight, the manuscript might not align with the broader biological focus typically expected by a journal like PLoS Biology.

Overall, the study presents a novel and interesting method for chemically inducing bacterial metabolites that may have beneficial effects on host organisms. However, the manuscript would benefit from a more transparent discussion of the role of CA in mammals and a stronger contextualization of the study within the broader field of microbiome-based therapeutics. The authors should consider focusing on the mechanistic novelty of the CA induction pathway, although this may require reassessing the manuscript's suitability for PLoS Biology.

Response: We thank the reviewer for finding our method novel and interesting. With the new mammalian results included in the revision, we believe that the manuscript has been further strengthened by demonstrating the beneficial effect of CA in mammals. Alongside the discovery of the non-canonical mechanism underlying low-dose-cephaloridine-induced CA production, we believe that the manuscript will be of a broad interest to the readers of PLOS Biology.

Reviewer 2

This is a review of the manuscript "Chemical Induction of Longevity-Promoting Colanic Acid in the Host's Microbiota" by Hu and colleagues for PLoS Biology. It previously has been shown that the polysaccharide Colanic Acid (CA) extends the lifespan of C. elegans and Drosophila. CA is produced by E. coli and related microbiota that can be found in the gut. In previous work, the authors used optogenetics to increase production of CA by gut microbiota, demonstrating that this was sufficient to induce longevity.

Here now the authors describe a chemical approach to induce CA production in the gut, by exposing animals to a low dose of cephaloridine. Cephaloridine is an antibiotic, but the dose used by the authors is below of what would be required to impair the bacterial growth. The authors show that this approach induces CA production in the gut of C. elegans and mice. Further, they show in C. elegans that such treatment is sufficient to induce longevity. Finally, they delineate the mechanism by which cephaloridine induces CA production in E. coli. They argue based on genetic experiments that cephaloridine acts on the bacterial membrane protein PBP1a which in turn relieves an inhibitory effect of the kinase ZraS on the RcsA/RcsB system. Then active RcsA/RcsB will activate transcription of the cps operon which encodes enzymes needed for CA production.

Overall, this is a solid story for a short report. The experiments have been well conducted and eventually reveal a new concept for how longevity could be promoted in vivo, using a drug that does not act on the organism of interest itself but that rather modulates the metabolism of its gut microbiota.

However, the following specific points should still be addressed:

Response: We appreciate that the reviewer finds our studies being solid, well conducted and revealing a new concept, and provides constructive suggestions.

1) You mention that CA is produced by E. coli and also other Enterobacteriaceae. Do you assume that the same mechanism of cepha-induced CA production exists in these other species, too? If so, what fraction of the human gut microbiota would actually be E. coli and what fraction those other Enterobacteriaceae? It would be valuable to clarify these points in the manuscript.

Response: We appreciate the reviewer's question and suggestion. *Enterobacteriaceae* are present in healthy human commensals with a small fraction [PMID: 26499895]. Within the *Enterobacteriaceae* family, *Escherichia* is the most dominant taxa in healthy humans [PMID: 20157339]. However, the presence of *cps* operon, which is responsible for CA production, in human commensal has not been previously analyzed. We have examined the presence of *cps* operon in the Unified Human Gastrointestinal Genome (UHGG) database. We detected the

existence of the *cps* operon in over 5000 genomes across 47 specifies, such as *Escherichia* (including *E. coli*)*, Salmonella, Enterobacter,* and *Citrobacter* (new Figure 1A). Furthermore, we compared the presence of the *cps* operon between centenarians and their elderly controls using two publicly available human centenarian gut microbiome datasets (PRJNA675598, PRJEB25514). We found that the read count of the *cps* operon was significantly higher in centenarians compared to controls in the Japanese gut microbiome dataset (PRJNA675598), which includes more than 100 individuals in each group ($p=0.015$, new Fig. 1B). A similar trend was also observed using the Italian gut microbiome dataset (PRJEB25514; $p=0.087$), although it did not reach statistical significance, likely due to the smaller sample size, with only ~20 individuals in each group (new Fig. 1C). These analyses revealed a potential correlation between the CA overproduction and human longevity. We have included these new analyses in the revised manuscript.

2) Line 162: You found before that a dose of 1.8 μM is optimal. Why did you then use here only higher doses of 4 and 8 μM? This is not clear to me.

Response: We thank the reviewer for raising this point. Using bacterial culture (*in vitro*), the optimal dose of 1.8 μg/mL was determined from the concentration-gradient assessment (new Figure 1F). However, when investigating the efficacy of cephaloridine treatment in mice (*in vivo*), determining the final drug concentration in the gut is more challenging. Therefore, we assessed a range of cephaloridine concentration at 2 μg/mL, 4 μg/mL and 8 μg/mL. We found that the induction of the *cps* operon occurred at both 2 μg/mL and 4 μg/mL, with 4 μg/mL showing a higher induction (new Figure 2G). One factor likely contributing to the difference in effective cephaloridine concentration between *in vivo* and *in vitro* conditions is luminal content dilution, which depends on the gut's volume and contents. To improve clarity, we have included the result from the 2 μg/mL treatment and discussed the dose-dependent response *in vivo*.

3) You primarily use growth curves to argue that the low Cepha doses don't give antibiotic effects. Given that in its function as an antibiotic Cepha targets the cell wall, it would be good to see directly that the cell remains unaffected by these low doses. Can you somehow directly monitor the condition and integrity of the cell wall?

Response: We appreciate the reviewer's question regarding the impact of low-dose cephaloridine on *E. coli* cell wall integrity. We interpret this concern as being closely tied to cell viability and function, as disruptions in cell wall integrity can lead to metabolic remodeling, reduced activity, and compromised viability. To address this, we applied stimulated Raman scattering (SRS) microscopy to directly assess single-cell metabolic activity by monitoring the incorporation of deuterium-labeled glucose. Glucose serves as a central carbon source for bacterial growth, and its incorporation provides a sensitive, quantitative readout of cellular metabolic activity. We believe this approach offers a more functionally relevant assessment than direct imaging of cell wall morphology, since the absence of observable structural changes does not necessarily indicate intact cellular function. In contrast, metabolic incorporation provides a direct and dynamic measure of cellular activity.

In this experiment, we cultured wild type *E. coli* overnight to an OD_{600} of 2.0, then diluted the culture 1:100 into fresh medium containing deuterium-labeled glucose, with or without low-dose

cephaloridine. We monitored deuterium incorporation at the single-cell level at 1-, 2-, 3-, 4-, and 6-hours post-inoculation. We found that the low-dose cephaloridine treatment did not alter deuterium-labeled glucose incorporation compared to the control across the 1–6-hour growth window (new Figure 1H, 1I, Supplementary 1B). This result indicates that *E. coli* metabolic activity and viability were not altered under this treatment condition.

4) Line 260: Your referral to Figure 4J does not make sense for me. Please double check.

Response: Thank the reviewer for pointing out this error. We have corrected this, and it is now Figure 4E in the revised manuscript.

5) Line 260: It is not clear to me why you consider ZraS to be responsible for the inhibition of CA production above 30°C and that Cepha releases this inhibitory effect. What is the evidence for that? This is a crucial part of the mechanism that should be addressed or clarified.

Response: We thank the reviewer for the suggestion to clarity this aspect of mechanism.

Our genetic analysis revealed that the CA level in the *zraS* deletion mutant was higher than in WT bacteria, even in the absence of the low-dose cephaloridine treatment (new Figure 4E). This finding suggests that ZraS normally suppresses CA production under baseline conditions. Furthermore, the low-dose cephaloridine treatment did not further elevate the CA level in the *zraS* deletion mutant (new Figure 4E), indicating that ZraS is required for mediating the CA-inducing effect of low-dose cephaloridine.

ZraP is a well-characterized downstream transcriptional target of ZraS activation and acts as a negative feedback regulator by inhibiting ZraS activity [PMID: 26438879, PMID: 30389436]. We found that *zraP* expression is up-regulated by low-dose cephaloridine in WT bacteria (new Figure 4D), further supporting the activation of ZraS under this condition. Taken together, these results support a model in which ZraS, in its inactive state, suppresses CA biosynthesis at 37°C, and low-dose cephaloridine activates ZraS, thereby relieving this suppression and promoting CA production.

We have revised the figure panel related to ZraS results as well as the text in the manuscript to clarity this mechanistic insight.

6) Likewise, how would binding of Cepha to PBP1a act on ZraS? Please give some better insight into this.

Response: We appreciate the reviewer's inquiry regarding how cephaloridine binding to PBP1a may influence ZraS activity. As discussed above, our genetic analysis suggests that low-dose cephaloridine activates ZraS, which in turn induces CA production. Although the exact molecular mechanism remains to be fully characterized, we speculate that cephaloridine binding to PBP1a causes a mild perturbation in the periplasmic environment, which is sensed by the periplasmic domain of ZraS, leading to its autophosphorylation. This activation likely promotes the phosphorylation of downstream regulator RcsAB, thereby up-regulating the *cps* operon and enhancing CA production.

In addition to its well-established role in the metal ion stress response, the ZraPSR two-component system has also been linked to β-lactam antibiotic sensitivity. A prior study showed that deletion of ZraP, a negative regulator of ZraS, increases resistance to cephalothin and cefluroxime, likely through ZraR-mediated transcriptional regulation of stress response genes [PMID: 30389436]. Although cephaloridine was not directly tested in that study, our findings show that low-dose cephaloridine transcriptionally up-regulates *zraP,* supporting the notion that it activates ZraS signaling.

Given that cephalosporin-class β-lactams, such as cephaloridine and cephalothin, primarily bind to PBP1a in *E. coli*, these findings collectively suggest that ZraS may specifically respond to PBP1a engagement by these compounds. We propose that cephaloridine binding to PBP1a may induce a mild periplasmic perturbation, which is sensed by ZraS, leading to its autophosphorylation. As a histidine kinase, ZraS may then phosphorylate downstream RcsAB to promote *cps* operon expression and CA production.

We have incorporated this discussion into the manuscript. Future studies will be required to completely elucidate the underlying molecular mechanism, which we believe is beyond the scope of the current manuscript.

7) What are the physiological benefits of the CA induction by Cepha in your mouse experiments? Could you see any? Please comment in the manuscript. If you saw any, they would be a great addition to this manuscript.

Response: We appreciate the reviewer's suggestions to investigate the physiological benefits of CA in mice. In response, we orally supplemented low-dose cephaloridine alongside wild-type MG1655 *E. coli* to 1-year-old mice for six months and collected both fecal samples and blood samples before and after the treatment. Our metagenomic analysis of fecal samples showed that the low-dose cephaloridine treatment did not decrease the microbiome richness, which supports the idea that cephaloridine does not result in bacterial lethality in the microbiota at this low-dose (new Supplementary Figure 2L-M). Furthermore, it is well established that LDL and insulin levels gradually increase with age in mice, due to aging-related metabolic dysfunctions. Consistently, we observed an age-related increase in LDL levels in male mice, whereas insulin levels increased in female mice between 12 and 18 months of age (new Figure 2H, 2I). Notably, these age-related increases were attenuated by the cephaloridine treatment (new Figure 2H, 2I). These new findings suggest cephaloridine-induced CA production mitigates age-related metabolic dysfunction in mice.

To further examine the possible role of CA induction in human longevity, we first examined the presence of *cps* operon, which is responsible for CA production, in the Unified Human Gastrointestinal Genome (UHGG) database. We detected the existence of the *cps* operon in over 5000 genomes across 47 specifies, such as *Escherichia* (including *E. coli*)*, Salmonella, Enterobacter* and *Citrobacter* (new Figure 1A). Furthermore, we compared the presence of the *cps* operon between centenarians and their elderly controls using two publicly available human centenarian gut microbiome datasets (PRJNA675598, PRJEB25514). We found that the read count of the *cps* operon was significantly higher in centenarians compared to controls in the

Japanese gut microbiome dataset (PRJNA675598), which includes more than 100 individuals in each group (*p=0.015*, new Fig. 1B). A similar trend was also observed using the Italian gut microbiome dataset (PRJEB25514; *p=0.087*), although it did not reach statistical significance, likely due to the smaller sample size, with only ~20 individuals in each group (new Fig. 1C). These analyses revealed a potential correlation between the CA overproduction and human longevity.

These new results have been included in the revised manuscript.

8) Line 301: What is the evidence that bacteria don't metabolize the Cepha? Is there a reference showing this?

Response: Thank the reviewer for raising this question. It is known that the genetic product from *ampC* in *E. coli*, as well as many *Enterobacteriaceae*, can metabolize cephaloridine and other cephalosporins [PMID: 19136439]. In wild-type *E. coli* MG1655, *ampC* expression is at a low level [PMID: 7010184], and the low-dose cephaloridine treatment did not induce its expression in our analysis (new Supplementary Figure 3). Based on this information, the wild-type MG1655 strain used in this study is unlikely to metabolize cephaloridine. However, we do not have direct evidence to confirm that cephaloridine is not metabolized in the mouse gut by other bacteria. We have included these references and added the discussion on this point (line 282-288).

Reviewer 3

Hu te al. present an intriguing investigation into the induction of colanic acid (CA) by E. coli both in culture and in vivo in the mouse gut. Previously, the group had identified CA as a lifespan-extending compound as part of a larger screen for microbial modulators of C. elegans aging. Here, they aim to use E. coli to deliver Colanic acid to the guts of mice.

The work is careful and well-considered—the authors discover new aspects of E. coli gene regulation and metabolism while finding some success in their overall synthetic biology / engineering goal. The methods they use are compelling, a mixture of E. coli bio-engineering and in vivo experimentation in nematodes and mice. However, the authors do not appear to provide direct evidence that they have achieved "chemical induction of longevity-promoting Colanic acid in the host's microbiota" in either C. elegans or mice. In C. elegans, the authors rely on lifespan as a proxy for CA production, but this proxy needs more validation (see point 1). In mice, the authors rely on a cleverly-engineered bacterial reporter for cps promoter activity which again needs additional validation. This reviewer does not think that direct measurement of CA induction in the gut is strictly required for the authors' results to be convincing—though such measurements would be helpful, definitive proof.

Response: We appreciate the reviewer's positive feedback, and we are glad that the reviewer finds the study intriguing, careful and well-considered. We agree that directly measuring CA induction in the gut would be helpful. However, such measurements are currently challenging due to technical difficulties associated with the complex chemical composition of the gut environment.

Major points

1. The authors show that treatment with CA at the CAI dosage extends C. elegans lifespan by 14%. This is a relatively small effect for nematode lifespan experiments—smaller than the reciprocal lifespan shortening the authors previously saw produced by Δlon and ΔHns mutants. What is the evidence that the lifespan extension produced by antibiotic treatment is specifically mediated by CA production? Could the cephaloridine be acting directly on some target in C. elegans to produce this small lifespan extension independently of its influence on E. coli?

Response: We appreciate the reviewer's question regarding the lifespan-extending effect of cephaloridine through inducing CA in bacteria. In our studies, we treated different bacterial mutants with low-dose cephaloridine and examined the effect on *C. elegans* lifespan. One of them is the deletion mutant of *rcsA* that controls the expression of *cps* operon in *E. coli*. We found that, low-dose cephaloridine failed to prolong *C. elegans* lifespan when applied to *E. coli rcsA* deletion mutant, in contrast to the wild-type strain (new Figure 3C). This result suggests that cephaloridine does not act directly on *C. elegans* but rather requires bacterial factors to mediate lifespan extension. If cephaloridine acted independently of bacteria, it would be expected to prolong lifespan even with the *rcsA* deletion mutant. Furthermore, we confirmed that *rcsA* deletion completely blocks cephaloridine-induced CA production in *E. coli* (new Figure 3B). These results support that low-dose cephaloridine acts through *rcsA* to induce CA production in *E. coli* and in turn extends lifespan in *C. elegans*.

We also appreciate the reviewer's observation that the lifespan-extending effect of cephaloridine is relatively weaker than those with the *Δlon and ΔHNS E. coli* deletion mutants. This difference is likely attributable to the relatively lower level of CA induction by cephaloridine compared to these genetic modifications.

2. The authors do not see an increase in RFP induction at the higher 8 ug/mL antibiotic dosage. The authors' favoured model seems to be that cephaloridine at this dose acts as an anti-microbial in vivo, unlike at 4 ug/mL. However, the authors provide no evidence for this. To clarify the missing effect at 8 ug/uL, the authors should provide some evidence that 4 ug/mL and 8 ug/mL dosages fall into distinct regimes in respect to anti-microbial activities. Otherwise, the reader is left wondering whether the experiments suffer from some technical issue—for example that the difference between 4 ug and 8 ug per mL results from inconsistent batch-to-batch variation and not a true dose-dependent qualitative difference in cephaloridine activity.

Response: We appreciate the reviewer's concern on the dose effect of cephaloridine. Cephaloridine-induced CA production is dose-dependent in both *in vitro* and *in vivo* conditions. Using *E. coli* culture (*in vitro*), the optimal dose of 1.8 μg/mL to induce CA production was determined from the concentration-gradient assessment (new Figure 1F). In this *in vitro* condition, it is technically possible to quantity CA levels using *E. coli* culture medium. However, when investigating the efficacy of cephaloridine treatment in mice (*in vivo*), determining the final drug concentration in the gut is more challenging. We were also unable to directly quantify CA levels using gut luminal content. To meet these challenges, we generated a fluorescent reporter *E. coli* strain to assess the induction of the *cps* operon in the mouse gut and analyzed a range of cephaloridine concentration at 2 μg/mL, 4 μg/mL and 8 μg/mL. We found that the induction of the *cps* operon occurred at both 2 μg/mL and 4 μg/mL, with 4 μg/mL showing a

higher induction (new Figure 2G). One factor likely contributing to the difference in effective cephaloridine concentration between *in vivo* and *in vitro* conditions is luminal content dilution, which depends on the gut's volume and contents. In the original submission, we didn't include the result from the 2 μg/mL treatment and instead focused on 4 μg/mL, as it provided the optimal induction. We agree with the reviewer that it is crucial to discuss the dose-dependent response *in vivo* and therefore added the result from the 2 μg/mL treatment in the revision.

In addition, for each treatment, at least three mice were analyzed as biological replicates, and all the treatments were repeated on different days. The ratio increase caused by cephaloridine was consistent, and no batch-to-batch variations were observed.

Minor points

1. In describing previous work, the authors state that "[CA] cannot be effectively delivered through the GI tract". Absent additional explanation, this fact seems to preclude the author's stated goal of using E. coli to deliver CA via the GI tract. The authors should give some more context or explanation. A citation would also help readers to understand the background for these statements.

Response: We thank the reviewer for pointing out the ambiguity of this statement. In our previous work, we attempted to supply CA-containing water to mice but found that the CA polymer is hydrolyzed into sugar monomers in a low-pH solution that mimics the stomach environment. An alternative approach would be to encapsulate purified CA to protect its integrity. However, this method presents challenges, including the large-scale preparation of CA power and difficulties in controlling the timing of compound release. Therefore, we investigated an approach to induce CA production from host gut microbiota and identified cephaloridine as a chemical inducer. We have revised the text to avoid confusion.

2. The authors state that the most effective CA-inducing concentration of cephaloridine is 1.8 ug/mL, which they claim is lower than a previously reported MIC value of 2 ug/mL. 1.8 does not seem to be meaningfully lower than 2. Do the authors have some statistical analysis to suggest the 0.2 ug/mL difference can be interpreted in this way? An straightforward solution would be for the authors to directly compare the CAI and MIC dosages in a single experiment under controlled conditions.

Response: We appreciate the reviewer's suggestion. MIC assessment is often condition-dependent, and reported MIC values of cephaloridine against *E. coli* vary in literature. The purpose of including published MICs was to provide a context for readers to assess the concentration used to induce CA in *E. coli,* not to imply that MIC values are relevant to the CA-inducing effect of cephaloridine. In our study, we provided direct evidence that cephaloridine at 1.8 μg/mL most effectively induces CA production *in vitro* without affecting bacteria growth or activity (new Figure 1F-I), confirming that its CA-inducing effect is not a result of bacteria growth inhibition. Since determining MIC values does not contribute to evaluating the CA-inducing effect of cephaloridine and would neither support nor contradict this conclusion, we have revised the manuscript and removed the discussion related to MIC values in this context.

3. The authors state that rtsB deletion inhibits bacterial growth in "M9 culture medium". The conventional recipe for this medium lacks both carbon and nitrogen sources. So growth wouldn't be expected by any E. coli strain, no?

Response: We appreciate the reviewer's question. We would like to clarity that we don't use M9 salt that lacks carbon and nitrogen sources. In M9 culture medium that we have used, glucose serves as the carbon source, while casamino amino acids provide nitrogen. In addition, $MgSO_4$, $CaCl_2$, $FeCl_2$, and Thiamine HCl are included as supplements. To avoid confusion, we have revised the text to "The *rstB* deletion mutant could not be evaluated in this analysis due to its impaired growth in M9 medium, even when supplemented with both carbon and nitrogen sources." (line 246-248)

4. The authors state that their CAI dosage is "unlikely to stimulate the general beta-lactan antibiotic stress response". The authors provide evidence for this, but the reader is left wondering why this observation is important. Can the authors explain a bit more?

Response: We appreciate the reviewer's comment and agree that this observation is not essential for understanding the CA-inducing effect of cephaloridine. However, we included this information to clarify that the CAI dosage of cephaloridine does not elicit a general β-lactam antibiotic stress response. This distinction supports that the observed induction of CA is not a secondary consequence of general antibiotic-mediated growth inhibition, but acts through a specific signaling mechanism. We have revised the text to explain this result more clearly (line 256-259).

5. The authors identify an 8% increase in GFP-to-RFP ratios from their in vivo experiments (Fig 2G), a smaller effect than the 20% they see in liquid culture (Fig 2E). Can the authors explain this difference?

Response: We appreciate the reviewer's question. Under *in vitro* condition, the ratio increased from 0.87 (vehicle) to 1.05 (cephaloridine), while under *in vivo* condition, it increased from 2.19 (vehicle) to 2.41 (cephaloridine). Thus, the ratio increases from base line—0.18 *in vitro* and 0.22 *in vivo*—are comparable. However, due to the higher baseline in the *in vivo* condition, the percentage of increase appears lower. In the revised manuscript, we have replaced 'Percentage of Increase' with 'Change from Base Line (GFP-to-RFP ratio)' to better present the results and have included raw numbers in the main text and figure legends.

6. It is great to see the authors learning something new about E. coli metabolism in the context of this larger engineering effort. How does the observed regulation of CA by ZraS mesh with the other known physiologic activities of ZraS? Some more perspective would be interesting if space allows.

Response: Thank the reviewer for the suggestion to add additional discussion regarding the known physiological activity of ZraS. In the revised manuscript, we have included the following discussion, "In addition to its well-established role in the metal ion stress response, the ZraPSR two-component system has also been linked to β-lactam antibiotic sensitivity [PMID: 30389436].

Within the ZraPSR system, ZraP is a transcriptional target activated downstream of ZraS, and functions as a negative feedback regulator, inhibiting ZraS. A previous study reported that deletion of ZraP increases resistance to cephalothin and cefuroxime, likely through ZraR-mediated transcriptional regulation of stress response genes [PMID: 30389436]. Although cephaloridine was not tested in that study, our findings show that cephaloridine up-regulates *zraP*, supporting the notion that it activates ZraS signaling. Given that cephalosporin-class β-lactams, such as cephaloridine and cephalothin, primarily bind to PBP1a in *E. coli,* these findings suggest that ZraS may specifically respond to PBP1a engagement by these compounds. We propose that cephaloridine binding to PBP1a may induce a mild periplasmic perturbation, which is sensed by ZraS, leading to its autophosphorylation. As a histidine kinase, ZraS may then phosphorylate downstream RcsAB to promote *cps* operon expression and CA production.” (line 295-308)
