# Supplementary material for: Chemical modulation of gut bacterial metabolism induces colanic acid and extends the lifespan of nematode and mammalian hosts
Source: PLoS Biol. 2025 Nov 11;23(11):e3002749. doi: 10.1371/journal.pbio.3002749 (PMC12604771; doi:10.1371/journal.pbio.3002749)
Supplement: Attachment — Submitted filename: Pointbypoint_PBIOLOGY-D-24-01970R2.pdf [file pbio.3002749.s012.pdf]

Reviewer 1:
Accept.

Response: We thank the reviewer for the acceptance.

Reviewer 2:
I am satisfied by the revisions made by the authors, with one minor exception:
Regarding my point 1), you responded: "We appreciate the reviewer's question and suggestion. Enterobacteriaceae are present in healthy human commensals with a small fraction [PMID: 26499895]. Within the Enterobacteriaceae family, Escherichia is the most dominant taxa in healthy humans [PMID: 20157339]. However, the presence of cps operon, which is responsible for CA production, in human commensal has not been previously analyzed. We have examined the presence of cps operon in the Unified Human Gastrointestinal Genome (UHGG) database. We detected the existence of the cps operon in over 5000 genomes across 47 species, such as Escherichia (including E. coli), Salmonella, Enterobacter, and Citrobacter (new Figure 1A). Furthermore, we compared the presence of the cps operon between centenarians and their elderly controls using two publicly available human centenarian gut microbiome datasets (PRJNA675598, PRJEB25514). We found that the read count of the cps operon was significantly higher in centenarians compared to controls in the Japanese gut microbiome dataset (PRJNA675598), which includes more than 100 individuals in each group (p=0.015, new Fig. 1B). A similar trend was also observed using the Italian gut microbiome dataset (PRJEB25514; p=0.087), although it did not reach statistical significance, likely due to the smaller sample size, with only ~20 individuals in each group (new Fig. 1C). These analyses revealed a potential correlation between the CA overproduction and human longevity. We have included these new analyses in the revised manuscript."

My follow-up: It's great to hear that the cps operon exists also beyond the Enterobacteriaceae family. However, can you please provide the readers with actual or at least approximate numbers on what fraction of human commensals tends to be Enterobacteriaceae and what fraction tend to be bacteria carrying the cps operon? Just saying "small franction" or "5000 genomes across 47 species" does not give me a true sense. I think that the readers would appreciate this information.

Response: We thank the reviewer for the thoughtful follow-up and the helpful suggestion to include quantitative information on the abundance of *Enterobacteriaceae* and cps-operon-harboring bacteria in the human gut microbiota.

According to a recent study [PMID: 38534413], members of the *Enterobacteriaceae* family typically comprise less than 1% of the healthy human gut microbiota. In our own analysis of publicly available metagenomic datasets, we found that *Enterobacteriaceae* accounted for approximately 1.43% of total reads in the Italian cohort (PRJEB25514) and 3.02% in the Japanese cohort (PRJNA675598).

To assess the prevalence of *cps*-operon-carrying bacteria more broadly, we examined the Unified Human Gastrointestinal Genome (UHGG) collection. In this database, 47 out of 4,744 species (approximately 0.99%) include at least one genome that carries the *cps* operon, corresponding to 5,578 genomes out of 289,232 total genomes. In the metagenomic datasets, we observed that an average of 9.16% of total reads in the Japanese cohort and 7.25% in the Italian cohort mapped to the representative *cps*-positive genomes of these 47 species.

We have added this quantitative information to the revised manuscript to improve clarity for the readers (attached below). We thank the reviewer again for this valuable suggestion.

*"To examine the relevance of the cps operon in the human gut microbiome, we have searched the United Human Gastrointestinal Genome (UHGG)[22] using DIAMOND[23]. In this database, 47 out of 4,744 species (approximately 0.99%) contain at least one genome that carries the cps operon, corresponding to 5,578 genomes out of 289,232 total genomes. These include 4598 in Escherichia, 466 in Salmonella, 341 in Enterobacter, 147 in Citrobacter, and 26 in other species (**Fig. 1A**).*

Next, we analyzed two publicly available metagenomic datasets of human centenarian gut microbiome (PRJNA675598, PRJEB25514)[24,25]. A recent study reported that members of the Enterobacteriaceae family typically comprise less than 1% of the healthy human gut microbiota [PMID: 38534413]. In our analysis, Enterobacteriaceae accounted for approximately 1.43% of total reads in the Italian cohort (PRJEB25514) and 3.02% in the Japanese cohort (PRJNA675598). We further compared the presence of the cps operon between centenarians and their elderly controls in these two cohorts. On average, 9.16% of total reads in the Japanese cohort and 7.25% in the Italian cohort mapped to the representative cps-positive genomes from 47 identified species."

<u>Reviewer 3:</u>

The authors have addressed all of my original concerns, which is very good.

Response: We appreciate the reviewer's positive feedback, and we are glad that our revisions have satisfactorily addressed the original concerns.

I have only one major concern with the revised manuscript: I find the new human microbiome sequence data to be entirely unconvincing. The difference between centenarians and control in the Italian cohort is not statistically significant, which many would interpret as supporting the opposite conclusion reached by the authors: that centenarians do not differ in the gut microbiome composition at this locus. The authors deviate from statistical best practices by speculating their negative result is "likely due to the smaller sample size, with only ~20 individuals in each group". This is not correct. The data demonstrates clearly that no large differences exist between the subgroups in the Italian cohort. Only differences small enough to be invisible when measured using 20 individuals might still exist despite the data presented. The best the authors can do here is perform a power calculation that estimates the minimum effect size identifiable from these 20 individuals—this minimum then sets the ceiling on the maximum difference that might exist. Would this small magnitude then retain the potential to be convincing? The authors have much more solid data elsewhere in their manuscript--it's unclear why the authors attempt to add further support to their claims using additional data lacking statistically significant effects.

Response: We thank the reviewer for the thoughtful comment regarding the statistical analysis. In response, we have carefully revisited the analysis of the Italian cohort.

In the revised manuscript, we initially applied a non-parametric Wilcoxon rank-sum test. However, upon further examination, we found that the data distribution is approximately normal after $\log_{10}$ transformation, as assessed using the Shapiro-Wilk test ($p > 0.05$). Given this, a parametric approach is more appropriate. We therefore re-analyzed the data using a two-sided Welch's *t*-test on the log-transformed values, which revealed a statistically significant difference between centenarians and elderly individuals in the Italian cohort ($p = 0.0436$). This contrasts with the previous *p* value ($p = 0.087$) and suggests that the earlier non-significance may have been influenced by the reduced power and conservative nature of the Wilcoxon test in small-sample settings.

To further contextualize these results, we conducted a formal power analysis to estimate the minimum detectable effect size (MDES) for the Italian cohort, which includes approximately 20 individuals per group. The analysis indicates that, at 80% power and $\alpha = 0.05$, the MDES is approximately Cohen's $d = 0.91$. As expected, only differences of large magnitude can be reliably detected given this sample size. In contrast, our observed effect size in the Italian cohort is Cohen's $d = 0.64$, which falls within the range typically interpreted as a moderate effect (d = 0.50 ~ 1.00). While this cohort is underpowered to detect small differences with high confidence, the observed effect remains meaningful and non-trivial. We have now included these results in the revised manuscript (attached below).

We agree with the reviewer that careful interpretation is essential when working with small sample sizes. In line with this, we have removed previous speculative language attributing the lack of significance solely "due to small sample size". We reframed the discussion more cautiously and present a more balanced interpretation rooted in effect size and power estimation. While not conclusive on their own, the consistent trends observed in both cohorts suggest a potential association between elevated *cps* operon levels and human longevity, and provide a rationale for the mechanistic studies described in the manuscript.

Even in the Japanese cohort, where the difference between centenarians and control does reach a permissive definition of statistical significance, the effect size is not stated but is clearly very small. For their data to be convincing, the authors would need to explain how this small statistical difference between groups reflects biologically meaningful difference, for example. Why should a small difference at this locus be expected to have any effect at all? Furthermore, an effect size this small would seem to be possibly generated by any of the various potential confounders that likely to exist between these two groups--do the authors try to control for diet, lifestyle, and age as potential confounders that might directly modulate cps frequencies? Doesn't it seem more likely that the measured difference in cps opron abundance result as the downstream effect of the many differences in lifestyle, diet, medication consumption, and aged gut microenvironment of centenarians compared to control populations? The likely causal model would then be (being old) -> (cps operon abundance), and not the authors favored model (cps operon abundance) -> (reaching old age).

Response: The reviewer is correct in noting that the difference in *cps* operon abundance between centenarians and elderly, while statistically significant (*t*-test $p = 0.0071$, analyzed using two-sided Welch's *t*-test based on normal distribution of the data), reflects a relatively small effect size (Cohen's $d = 0.37$). This corresponds to a modest effect ($d = 0.21 \sim 0.50$); however, the large sample size (~110 individuals per group) provided sufficient statistical power to detect such differences. In the revised manuscript, we now clearly report the effect size and avoid overstating the biological relevance of this result.

We also agree with the reviewer that differences in lifestyle, diet, medications, or other environmental factors may contribute to the observed variation in *cps* operon abundance. Since these publicly available datasets do not provide sufficient metadata for covariate adjustment, we explicitly acknowledge this limitation in the revised text. As suggested by the reviewer, we have also added discussion on potential confounding factors such as age-related lifestyle, medication, and dietary differences, and acknowledge that the observed associations might reflect downstream consequences of aging, rather than the direct causal contributors to longevity (attached below).

My recommendation to the authors would be to remove all the human data, as it detracts from an otherwise convincing model organism project. Alternatively, the authors should adhere to statistical best practices and conclude that they observe a very small difference (stating the effect size) in one out of two cohorts of centenarians, while addressing the potential for any such differences to result from a variety of potential confounds.

Response: While we agree that the human cohort data is not central to the mechanistic focus of the manuscript, we believe that it adds translational value by illustrating modest, but real-world associations observed in natural populations. In addition, operon-level analyses of this kind have not been broadly applied to gut microbiota genome datasets, and we hope our approach may inspire further studies that yield new insights into the microbiome's influence on host physiology and pathology. Importantly, in the revised manuscript, we have substantially tempered the conclusions drawn from these data. We now clearly report effect sizes, acknowledge statistical limitations, and explicitly discuss the potential for confounding factors.

*"In the Japanese cohort, which includes over 100 individuals per group, we observed a statistically significant difference in cps operon abundance between centenarians and controls (**Fig. 1B**, Welch's t-test, p = 0.0071), with a small-to-moderate effect size (Cohen's d = 0.37). In the Italian cohort, which includes approximately 20 individuals per group, a statistically significant difference was also observed (**Fig. 1C**, Welch's t-test, p = 0.0436), with a moderate effect size (Cohen's d = 0.64). Notably, a post hoc power analysis based on this effect size suggests that approximately 39 individuals per group would be required to achieve 80% power at α = 0.05, indicating that the Italian dataset may have been underpowered in the original analysis. Despite this limitation, these findings collectively support a small-to-modest increase of cps operon abundance in centenarian individuals. Unfortunately, neither cohort studies included information on individual diet, medication use, or lifestyle factors, preventing us from controlling these potential confounding variables. Moreover, this association analysis cannot clarity whether the increased cps operon abundance contributes to longevity or is simply a consequence of advanced age in centenarians."*
